# Hypothermia in Preterm Infants in the First Hours after Birth: Occurrence, Course and Risk Factors

**DOI:** 10.1371/journal.pone.0164817

**Published:** 2016-11-03

**Authors:** Arenda Mank, Henriëtte A. van Zanten, Michael P. Meyer, Steffen Pauws, Enrico Lopriore, Arjan B. te Pas

**Affiliations:** 1 Division of Neonatology, Department of Pediatrics, Leiden University Medical Center, Leiden, the Netherlands; 2 Division of Neonatology, Department of Pediatrics, Middlemore Hospital, Auckland, New Zealand; 3 Philips Research - Healthcare, Eindhoven, The Netherlands; Hopital Robert Debre, FRANCE

## Abstract

**Background:**

Hypothermia is associated with increased morbidity and mortality rates. Preterm infants frequently have hypothermia when they are admitted to the NICU, but there is no data on the occurrence of hypothermia during the first hours after admission.

**Objective:**

To investigate the occurrence of hypothermia in preterm infants in the first three hours of admission and to identify risk factors.

**Methods:**

Infants < 32 weeks of gestation included in a randomized trial with admission temperature as primary outcome were retrospectively analyzed for the occurrence of hypothermia (< 36.5°C) in the first three hours after admission. Risk factors were identified using linear regression analysis and logistic regression.

**Results:**

In total 80 infants were included with a median (IQR) gestational age at birth of 29 (27–30) weeks. In 93% of the infants hypothermia occurred in the first three hours after admission. The median (IQR) duration of hypothermia was 101 (34–162) minutes, of which 24 (7–52) minutes the hypothermia was mild, 45 (4–111) minutes moderate, severe hypothermia hardly occurred. Gestational age and the occurrence of hypothermia at birth were independent risk factors for the occurrence of moderate and severe hypothermia and significantly correlated with duration of hypothermia.

**Conclusions:**

Hypothermia occurred often and for a long period in preterm infants in the first three hours of life, low gestational age and admission temperature were independent risk factors

## Introduction

The large body surface area in relation to weight and the relative lack of subcutaneous fat make preterm infants at risk for hypothermia (body temperature below 36.5°C), especially in the first few hours after birth [1]. Laptook et al reported that admission hypothermia occurred in 47% of the infants ≤28 weeks gestational age [2]. Prevention of hypothermia in preterm infants is essential as studies have shown that neonatal hypothermia is associated with increased morbidity and mortality rates [2–8]. Hypothermia is also a risk factor for arrhythmia, bleeding, thrombosis, sepsis [9] and intraventricular hemorrhage (IVH) [10].

Interventions to minimize heat loss in preterm infants at birth are recommended in the Neonatal Resuscitation Program, including the use of plastic bags or plastic wrapping under a radiant heater [9] and are now standard care. However, despite these measures, nearly half of the preterm infants have hypothermia at admission [2]. We recently reported that the rate of hypothermia at admission is lower when heated and humidified air is used during resuscitation at birth, but the rate of hypothermia remained high [11]. Although measures are taken to prevent hypothermia during admission by using a (servo-controlled) incubator, there is no data on the occurrence of hypothermia during the first hours after admission. Procedures (intubation and umbilical line catheterization) are most often performed in the first hours and can potentially lead to hypothermia.

The aim of this study is to determine the occurrence and duration of hypothermia in infants < 32 weeks gestational age at birth during the first three hours after arrival at our neonatal intensive care unit (NICU) and to identify risk factors.

## Methods

In this retrospective study we included all preterm infants (gestational age at birth < 32 weeks) participating in the HEAT trial [11] at the Leiden University Medical Center from February 2008 to May 2009. In the HEAT trial the use of heated humidified air was compared with cold dry air during respiratory support of infants < 32 weeks gestational age at and admission temperature was taken as primary outcome. [11] Since this was a retrospective study, it did not need to comply with the Dutch law on Medical Research in Humans, and the Research Ethics Committee stated it had no objection.

Rectal and axillary temperature is routinely measured in all neonates admitted to our neonatal nursery. During admission skin temperature was continuously measured by a skin temperature probe (Philips skin temperature probe; Philips Medizin System, Boeblingen, Germany) and recorded every minute in the Patient Data Management System (PDMS) (Metavision; IMDsoft, Tel Aviv, Israel). Infants were excluded if skin temperature was not available in PDMS. Outliers and errors in temperature data points were removed from the database and defined as a decrease in temperature by 1°C or more within 1 minute. The occurrence of hypothermia during the first three hours after admission to the NICU was taken as primary outcome, but also the duration was noted. Normothermia was defined as skin temperature of 36.5°C to 37.5°C, hypothermia was defined as <36.5°C (mild hypothermia: 36.0°C to 36.4°C, moderate hypothermia: 34.0 to 35.9°C and severe hypothermia: <34.0°C) and hyperthermia was defined as >37.5°C. Duration of hypothermia was defined as the sum of hypothermic minutes in the first three hours after admission. The following parameters were expected to be risk factors and noted: Gestational age (GA) at birth, birth weight (BW), small for gestational age (< 10^th^ percentile), placement of umbilical lines, endotracheal intubation in the first three hours and the occurrence of hypothermia at admission (rectal T < 36.5°C).

After infants were stabilized after birth they were brought to the NICU in a transport incubator (NICU Roadrunner) (Maastricht Instruments, Maastricht, The Netherlands). Immediately after arrival they were placed in an incubator (Caleo^®^, Dräger, Zoetermeer, The Netherlands). The incubators were on air control mode with alert limits <36.5°C and >37.3°C on the Philips monitor. Initial temperature was set at 34–36°C and humidification 60%-70% depending on gestational age. The wrap was taken off after placement in the incubator in the unit. For procedures (i.e. intubation, catheterization), where it was needed to open the incubator, the infants where covered with a transparent plastic wrap while the procedure was performed under a radiant heater.

### Statistics

SPSS 20.0 for Windows was used for all analyses. Data are presented as median (interquartile range), mean (SD), or number (percentage). Outcome parameters were compared by using Student t test for parametric comparisons, the Mann-Whitney U test for nonparametric comparisons for continuous variables and the x^2^ test for categorical variables. For correlation we used Pearson r, risk factors were adjusted for other risk factors using linear regression analysis and the logistic regression analysis. Considering the very strong relationship between GA and BW only GA is used as a factor in the regression analysis. Reported P values were 2-sided, p <0.05 was considered statistically significant.

## Results

The charts of all 107 infants included in the trial in Leiden were reviewed, 27 infants needed to be excluded for this analysis because skin temperature was not available in PDMS. Thus, 80 patients could be included in this study (basic characteristics see Table 1) and for these patients in total 98% of the minute data points in temperature could be retrieved, of which 2% were outliers and excluded for analysis.

**Table 1:** Baseline characteristics.

Characteristic	All infants (n = 80)
Males, n (%)	44 (55)
Apgar score at 5 min, median (IQR)	8 (7–9)
Singleton, n (%)	33 (42)
IUGR, n (%)	19 (24)
Cesarean delivery, n (%)	44 (55)
Gestational age, median (IQR)	29 (27–30) weeks
Birth weight, median (IQR)	1151 (891–1396) grams
**Procedures, n (%)**	36 (45)
Umbilical lines, n (%)	34 (43)
Endotracheal intubation, n (%)	11 (14)
Hypothermia at admission, n (%)	24 (30)

IUGR indicates intrauterine growth retardation defined as growth below the 10th percentile

### Hypothermia

In 93% (74/80) of the infants hypothermia occurred in the first three hours after admission, in 76% (61/80) of the infants hypothermia lasted more than 30 minutes. In all 80 patients, the median (IQR) duration of hypothermia in the first three hours after admission was 101 (34–162) minutes, of which 24 (7–52) minutes hypothermia was mild, 45 (4–111) minutes moderate and 0 (0–0) minutes severe (range = 0–148 minutes).

In the normothermic group at admission (n = 56), most infants (n = 50, 89%) became hypothermic in the first three hours. Of these 50 infants, 40% (n = 20) had umbilical lines placed and 16% (n = 8) was intubated in the first three hours. The six infants (11%) who did not become hypothermic in the first three hours, had no umbilical lines and were not intubated in the first three hours.

### Hyperthermia

In 23% of the infants mild hyperthermia occurred in the first three hours after admission, in 13% this occurred for more than 30 minutes. The median duration of mild hyperthermia was 0 (0–0) minutes (range = 0–110 minutes).

### Factors associated with duration of hypothermia

**Fig 1:**
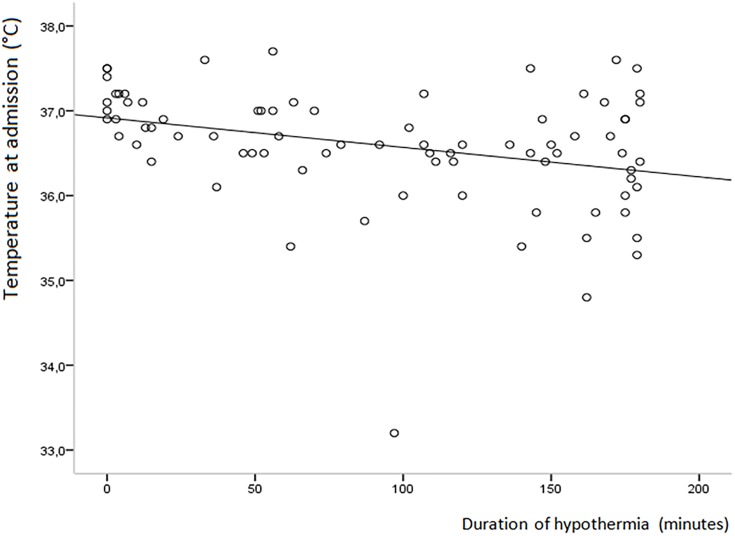
Correlation between temperature at admission and duration of hypothermia.

There was a moderate, positive correlation between the duration of hypothermia in the first three hours and hypothermia at admission (r = 0.37; p< 0.001) and placing umbilical lines (r = 0.40; p< 0.001) and a medium, negative correlation with the temperature at admission (r = -0.32; p< 0.05) (Fig 1), gestational age (r = -0.45, p< 0.001) and birth weight (r = -0.47, p< 0.001). SGA and the need for intubation in the first three hours were not correlated with the duration of hypothermia. When adjusted for other risk factors only gestational age (p< 0.01), and hypothermia at admission (p< 0.01) remained significantly correlated, but not placing umbilical lines.

Infants with moderate to severe hypothermia > 30 minutes after admission had a lower birth weight (1070 (288) vs 1322 (368) grams; p< 0.01), had a lower gestational age (27.7 (2.0) vs 29.0 (1.6) weeks; p< 0.01), were more often hypothermic at admission (43% vs 12%; p< 0.01) and were more likely to have umbilical lines placed in the first three hours after admission (57% vs 21%; p< 0.01) when compared to the infants where this did not occur. Intubation in the first three hours (19% vs 6%; ns) and SGA (23% vs 24%; ns) were not significantly different. When adjusting for other risk factors using logistic regression analysis only hypothermia at admission (p< 0.05; OR 4.6 (1.3–16.5) and GA at birth (p< 0.05; OR 0.74 (0.55–0.99)) remained significantly associated with moderate to severe hypothermia > 30 min (Table 2).

**Table 2:** Risk factors for moderate to severe hypothermia > 30 min.

Factor	Mod/sev hypothermia (n = 47)	No Mod/sev hypothermia (n = 33)	Univariate analysis (p)	Multivariate Analysis (p)	OR (95%CI)
Birth weight, mean (SD)	1070 (288) grams	1322 (368)	< 0.01		
Gestational age, mean (SD)	27.7 (2.0) weeks	29.0 (1.6)	< 0.01	0.05	0.74 (0.55–0.99)
Hypothermia at admission, n (%)	20 (43)	4 (12)	< 0.01	< 0.05	4.6 (1.3–16.6)
IUGR, n (%)	11 (23)	8 (24)	ns	ns	1.2 (0.4–4.2)
Umbilical lines < 3 hours, n (%)	27 (57)	7 (21)	< 0.01	ns	2.7 (0.8–8.7)
Intubation < 3 hours, n (%)	9 (19)	2 (6)	ns	ns	2.2 (0.4–13.0)

### Course of temperature

**Fig 2:**
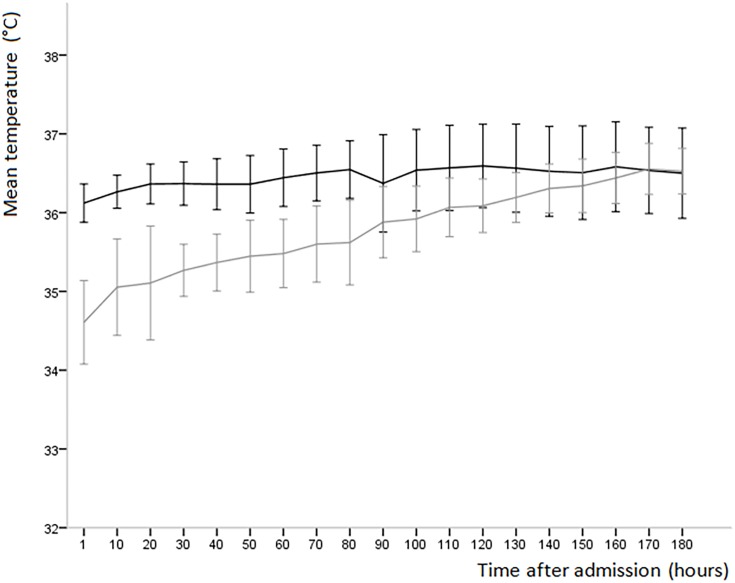
Mean temperature every 10^th^ minute (95% CI) of infants with hypothermia at admission (gray) and infants without hypothermia at admission (black) in the first three hours after admission.

Infants with hypothermia at admission had a significantly lower temperature until 80 minutes after birth (p< 0.05)when compared to infants without hypothermia at admission (Fig 2). Infants with an umbilical line had a significantly lower temperature (p< 0.05) for the entire first three hours after admission.

## Discussion

In this study we observed that preterm infants admitted to the NICU often have hypothermia in the first three hours after admission, which is mostly mild to moderate. When present, in many infants hypothermia lasted for a long period. Gestational age, hypothermia at admission, umbilical catheterization and intubation are all inter-related and, increase the risk for hypothermia. Lower gestational age at birth and hypothermia at admission remained independent risk factors for hypothermia in the first three hours. Indeed, infants with hypothermia at admission not only have a 5 times higher risk for hypothermia in the first three hours after admission, but the hypothermia also lasted longer when compared to infants with hypothermia during the first hours but normal temperature at admission. Although mostly mild to moderate hypothermia occurred, it is known to increase the risk for IVH and the occurrence and severity for RDS, which often occur in the first day of life [2, 4–6, 10, 12].

This is one of the few studies describing hypothermia in the first hours after admission. Most studies have focused on the incidence, prevention and risk factors of hypothermia at admission [2, 11–13]. Similar to our study, Laptook et al. reported an association between birth weight and hypothermia [2]. In contrast, Laptook also reported that intubation in the delivery room was a risk factor for hypothermia at admission, while we observed that intubation in the unit was not significantly associated with hypothermia after admission. It is possible that the intubation in the unit was in a more controlled circumstances and every measure to prevent cooling can be taken. Another explanation for finding no significant effect of intubation on hypothermia is that the sample size was too small. In a randomized study of preterm infants <33 weeks which compared radiant warmer and incubator care, temperatures in the incubator group were significantly lower in the first 2 hours after admission (mean (SD) 35.9 (0.3)°C vs 36.6 (0.8)°C; p = 0.007) although admission temperatures were similar [14]. Almost 30% of those in the incubator group had a recorded temperature below 36°C in the first 2 hours. In 20% of cases a temperature below 36°C was recorded between 2-4hrs after admission. Significant factors associated with low early temperatures were the type of device used (warmer or incubator) and birth weight. This does raise the question of whether a period of radiant warmer care to allow re-warming, using a higher set temperature for the incubator in hypothermic infants and using the incubator in skin servo-control mode may be beneficial.

In the present study, hypothermia occurred often and for a long period in the first three hours after admission to the NICU. In 93% of the infants hypothermia was present in the first three hours after admission and the median (IQR) duration of hypothermia was 101 (34–162) minutes. We decided to study the first three hours as we hypothesized that most procedures would then take place and could potentially lead to hypothermia. However, after adjustment for other risk factors both procedures (intubation and umbilical catheterization) were no longer significantly associated with the duration of hypothermia and whether hypothermia lasted longer than half an hour. In contrast, hypothermia at admission appeared to be the largest risk factor for hypothermia later on. It is logical that infants who have a low temperature at birth need more time to reach a normal temperature in contrast to infants without hypothermia at admission. Preventing hypothermia in the delivery room could then also lead to a significant reduction in hypothermia during admission.

This study was retrospective and missing data could have been a confounding factor. Not in all infants the temperature could be retrieved for the entire three hours. However, this probably had little influence as the proportion of time for which no data were available was only 4%. The duration of intubation and umbilical line procedures was not registered in PDMS and it is likely that hypothermia is associated with the duration and not with undergoing procedure itself. Also, for this study only skin temperature measurements were available and it is possible that temperature measured on the skin is lower than measured rectally. However, Helder et al observed only a small difference of skin-rectal temperature of—0.27 (-0.53–0.03)°C when measured on the abdomen and 0.03 (-0.23–0.29)°C when measured on the back. [15]

In conclusion, this retrospective study showed a high incidence of hypothermia in the first three hours after admission to the NICU. Gestational age at birth and hypothermia at admission were independent risk factors for the occurrence of hypothermia in the first hours during admission. Hypothermia occurred more often and it took infants longer to reach normothermia when they were admitted with hypothermia. Hypothermia will increase the risks in morbidity and mortality and caregivers should be aware of this and take all precautions to prevent hypothermia, not only in the delivery room, but also in the first hours after admission.
